# Japanese *Papilio* butterflies puddle using Na^+^ detected by contact chemosensilla in the proboscis

**DOI:** 10.1007/s00114-012-0976-3

**Published:** 2012-11-09

**Authors:** Takashi A. Inoue, Tamako Hata, Kiyoshi Asaoka, Tetsuo Ito, Kinuko Niihara, Hiroshi Hagiya, Fumio Yokohari

**Affiliations:** 1 National Institute of Agrobiological Sciences, Ôwashi 1-2, Tsukuba, Ibaraki 305-8634 Japan; 2 Kibogaoka 34-19, Asahi-ku, Yokohama, Kanagawa 241-0624 Japan; 3 Department of Natural Sciences, Faculty of Knowledge Engineering, Tokyo City University, Tamazutsumi, Setagaya-ku, Tokyo, 158-8557 Japan; 4 Division of Biology, Department of Earth System Science, Faculty of Science, Fukuoka University, Nanakuma 8-19-1, Jonan-ku, Fukuoka, Fukuoka 814-0180 Japan

**Keywords:** Contact chemosensillum, Metal ions, *Papilio*, Proboscis, Puddling, Tip recording

## Abstract

Many butterflies acquire nutrients from non-nectar sources such as puddles. To better understand how male *Papilio* butterflies identify suitable sites for puddling, we used behavioral and electrophysiological methods to examine the responses of Japanese *Papilio* butterflies to Na^+^, K^+^, Ca^2+^, and Mg^2+^. Based on behavioral analyses, these butterflies preferred a 10-mM Na^+^ solution to K^+^, Ca^2+^, and Mg^2+^ solutions of the same concentration and among a tested range of 1 mM to 1 M NaCl. We also measured the ion concentrations of solutions sampled from puddling sites in the field. Na^+^ concentrations of the samples were up to 6 mM, slightly lower than that preferred by butterflies in the behavioral experiments. Butterflies that sipped the 10 mM Na^+^ solution from the experimental trays did not continue to puddle on the ground. Additionally, butterflies puddled at sites where the concentrations of K^+^, Ca^2+^, and/or Mg^2+^ were higher than that of Na^+^. This suggests that K^+^, Ca^2+^, and Mg^2+^ do not interfere with the detection of Na^+^ by the *Papilio* butterfly. Using an electrophysiological method, tip recordings, receptor neurons in contact chemosensilla inside the proboscis evoked regularly firing impulses to 1, 10, and 100 mM NaCl solutions but not to CaCl_2_ or MgCl_2_. The dose–response patterns to the NaCl solutions were different among the neurons, which were classified into three types. These results showed that Japanese *Papilio* butterflies puddle using Na^+^ detected by the contact chemosensilla in the proboscis, which measure its concentration.

**Electronic supplementary material:**

The online version of this article (doi:10.1007/s00114-012-0976-3) contains supplementary material, which is available to authorized users.

## Introduction

Males of many butterfly and some moth species often land on damp ground to sip water solutions. This behavior is well known among lepidopterologists and naturalists. Norris (1935), who summarized the feeding habits of the adult Heteroneura (a suborder of Lepidoptera), called this behavior “water-drinking.” Arms et al. (1974) renamed this behavior “puddling” and included sipping behavior from dung and carrion in this category. However, in Japan, sipping behavior from dung and carrion is rare, whereas sipping behavior from puddles or small water streams, especially performed by Papilioninae and Pirinae butterflies, is common. This sight is one of the spectacles of tropical and sub-tropical areas, e.g., Japan, Southeast Asia, and Central and South America (supplemental pictures). When performing this behavior, butterflies and moths simultaneously sip and excrete water solutions as urine (e.g., Unno 2009). As most of the sites where puddling is performed appear to contain no nutritional components, many lepidopterologists have struggled to identify the purpose or role of puddling behavior.

This issue was studied independently with behavioral field studies, laboratory experiments on the proboscis reflex. Arms et al. (1974) first examined puddling behavior and observed in field experiments that *Papilio glaucus* preferred a 10-mM Na^+^ solution. Adler and Pearson (1980) measured the amounts of Na^+^ and K^+^ in the bodies of *Pieris rapae* and found that males preferred Na^+^ solutions and consumed more Na^+^ than did females. Based on these results, they concluded that male *P*. *rapae* butterflies puddled to obtain Na^+^. Beck et al. (1999) examined the puddling behavior of *Papilio helenus* and *Papilio memnon* in Borneo (Malaysia) and reported that these butterflies were also attracted to NaCl solutions. Boggs and Jackson (1991) examined the proportion of sex and age classes caught puddling versus feeding from flowers, using a Nymphalid butterfly, *Speyeria mormonia*. They described sexual differentiation in puddling activity, with males exhibiting a higher ratio of captures at puddling versus flowers, and females with damp wings and dry-but-undamaged wings spending less time puddling than older butterflies of either sex. Scriber and Ayres (1988) reported puddling in *P*. *glaucus* females and also mentioned that *P*. *glaucus canadensis* males “puddled” on bird guano. More recently, Boggs and Dau (2004) performed natural substrate puddling observations and a sand tray experiment and measured Na^+^ concentrations in the locations where butterflies puddled. They reported that 16 species of butterfly, including one *Papilio machaon* group species, more frequently visited carnivore dung and/or mud than herbivore dung and preferred 0.001 and 0.01 M Na^+^. The role of ingested Na^+^ in butterflies and moths has been proposed to be related to nuptial gifts (e.g., Lederhouse et al. 1990; Pivnick and McNeil 1987).

Independently of the puddling question, some researchers have examined the sensitivities of butterflies to some ions using the proboscis extension reflex by stimulating tarsi with taste solutions. Minnich (1922) and Weis (1930) demonstrated that the legs of *Vanessa atalanta* were sensitive to Na^+^. Kitaoka (1950) reported that *Vanessa indica* also responded to Na^+^ but not to Li^+^, K^+^, Mg^2+^, Ca^2+^, or Ba^2+^. Kuwabara (1951) examined of Na_*n*_X^*n*−^ reception in the legs of *V*. *indica* and found that sensitivity to Na^+^ was not affected by anion species. Kusano (1968) investigated *P*. *rapae* and 11 other Japanese butterfly species including *P*. *machaon* and *Papilio xuthus* and found that male pierid and hesperid butterflies had greater sensitivity to Na^+^ than did females. Additionally, Takeda (1961) examined contact chemosensilla on the ventral and lateral sides of the fifth tarsomeres of the mid and hind legs of *V*. *indica* using an electrophysiological method, tip recording, and found that large spikes occurred in response to NaCl as well as sucrose in some ventral hairs.

As described above, Na^+^ intake from puddling behavior is well known, and the Na^+^ detection system of butterflies is present at least on tarsi. We believe that the presence or absence of sipping is important when deciphering the relationship between puddling behavior and taste sensitivity as a means of evaluating the role of the puddling behavior. We have often observed that *Papilio* butterflies search for puddling points by hovering and locate puddling sites by touching water solutions with the tip of their proboscises but not with their legs (e.g., Unno 2009). Thus, we hypothesized that butterflies detect Na^+^ in water via contact with chemosensilla inside the proboscis.

The butterfly proboscis is formed from a pair of right and left galeae, and small hairs are present on the inside wall of the galeae (Eastham and Eassa 1955; Sellier 1975; Krenn 1998). Although the roles of these structures have not been examined thoroughly, we recently succeeded in recording impulse activity from these structures in response to sucrose stimuli in *P*. *xuthus* (Inoue et al. 2009). We showed that *Papilio* butterflies had no apparent taste sensilla on the outside of the proboscis; thus, the structures inside the proboscis must function as taste sensilla for sugar intake from flower nectar.

In the present study, to examine the relationship between preferences in puddling behavior and the electrophysiological response to metal ions of proboscis sensilla, we performed the following three experiments. First, we scored puddling behavior responses in eight different species of Japanese *Papilio* butterflies in the field and greenhouse to four major metal ions, Na^+^, K^+^, Ca^2+^, and Mg^2+^, using the effects of the anions on Na^+^ detection and Na^+^ concentration. Second, to determine whether the results of the behavioral experiments could be corroborated by observations on natural puddling sites, we collected water samples where butterflies puddled and analyzed the concentrations of the four cations. Third, we recorded impulse responses to various salt solutions from the taste sensilla inside the proboscis. Based on the findings obtained from these experiments, we discuss the features of puddling in butterflies and the association between the sensory reception of Na^+^ and puddling.

## Materials and methods

### Morphology

To correctly identify the sensilla in subsequent electrophysiological experiments, the interior of the proboscis of male *Papilio bianor* and male *Papilio protenor* was examined with a scanning electron microscope (SEM: JSM-6301 F, JEOL Co., Ltd., Tokyo, Japan). These species of butterflies were selected as our experimental animals for the following reasons: these species are very common in Japan, they are often observed to engage in puddling behavior, and *P*. *bianor* and *P*. *protenor* are representative species of the subgenera *Achillides* and *Menelaides*, respectively. In this experiment, we used summer-form butterflies that were standard in size and had well-coiled proboscises. These specimens were previously dried without any special treatment.

The proboscis was first cut out at its proximal end with scissors. As the sensilla are present on the inner surfaces of the pair of galeae that form the proboscis, they must be exposed to be made observable. The proboscises were attached right side down to the metal block platform using adhesive tape. Then, they were separated into two galeae using forceps, and the left galea was removed and coated with gold in an ion sputter (Sanyu Denshi, SC-701). The specimen was observed using a SEM, and the SEM images were digitally recorded. The number of sensilla inside the proboscis of each specimen was quantified based on the SEM digital images. Additionally, the number of these sensilla and the extended proboscis length were also measured under a binocular microscope (Leica MZ125) prior to the electrophysiological experiment in these two species and in *P*. *xuthus*.

### Behavioral experiments

To obtain behavioral data that we could compare with the electrophysiological results, we performed behavioral experiments in the field using Japanese *Papilio* butterflies and a method modified from Arms et al. (1974). In Japan, puddling behaviors can typically be observed for less than 1 week at each site. Additionally, the numbers of puddling butterflies are affected by climatic and geological conditions; therefore, we performed the experiments in many open fields in various districts throughout the Japanese mainland where *Papilio* butterflies are often observed to puddle. The experiments were performed almost every week from April to October during 2007–2011. We also performed the same experiments in two different greenhouses, at the Toyosato Insect Museum Green House, Tsukuba, and Adachi Park of Living Things, Tokyo.

Ten semitransparent trays made of polyethylene (330 × 220 × 45 mm) were prepared for this experiment. Each tray was filled with 2.2 L of sand (Kawasuna™, Tachikawa Heiwa Nouen, Kanuma, Tochigi, Japan) that had been washed with at least 10 L of Milli-Q water (Millipore, Billerica, MA, USA) and freeze dried. Ten different metal ion solutions were made, i.e., 10 mM NaCl, 10 mM KCl, 10 mM CaCl_2_, 10 mM MgCl_2_, 1 mM NaCl, 100 mM NaCl, 1 M NaCl, 10 mM NaNO_2_, 10 mM NaHCO_3_, and 10 mM NaH_2_PO_4_. A total of 3 dl of each solution was poured into a given tray to saturate the dried sand within the tray. These trays were rinsed with 1.5 L of the corresponding solution after every use to minimize contamination by foreign substances and to avoid fluctuations in ion concentrations, i.e., changes in concentration due to evaporation, elution from broken sand, and dilution by wash water.

These trays were placed at specific experimental sites near locations where *Papilio* butterflies had already been observed to puddle. In most trials, all 10 trays were used. Trays containing the 10 mM Na^+^ solution were always included, even in experiments in which only a few trays were used. As in previous experiments, a dead male of *P*. *xuthus*, *Papilio maackii*, and/or *P*. *protenor* was pinned on each tray as a decoy to orient butterflies to the trays (Arms et al. 1974; Beck et al. 1999). All trays placed at an experimental site were marked with decoys of the species or combination of species selected based on the *Papilio* species that were puddling there at the time.

Experiments were mainly performed between 08:30 and 11:30, which is when Japanese *Papilio* butterflies frequently puddle. To avoid any positional effects of the trays on the presence of butterflies at a given tray, tray position was changed every 5 or 15 min. Some naturalists have suggested that the existence of “living” puddling butterflies might induce other butterflies to want to puddle; actually, we often observed that quite young individuals followed slightly older individuals and landed on our trays with them. Therefore, butterflies that continued to puddle in the moved trays were taken out of the trays and allowed to fly away.

The behaviors of all *Papilio* butterflies that visited our experimental sites were recorded and scored as follows: Score 1, a butterfly lands on one of the trays, tastes the solution, and then immediately takes off; Score 2, a butterfly lands on one of the trays, tastes the solution, sips the solution for a few seconds while its wings are fluttering, and then takes off; Score 3, a butterfly lands on one of the trays, stops fluttering its wings, and sips the solution for longer than 10 s. Some butterflies had different behavioral scores on the same tray in sequential trials. In such cases, the score during the last trial was adopted.

### Ion concentration measurements at puddling sites

Fluids at the puddling sites were collected as described below. When the fluids were on paved roads or mud, they were aspirated with disposable pipettes or were soaked into absorbent paper (KIMWIPE®, Nippon Paper Crecia Co., Ltd). When the fluids were absorbed by a layer of mud or sand, the surface of the layer was scraped away over a 50 × 50 × 5-mm area. Each sample was bottled in a 15- or 50-ml polyethylene conical tube (BLUE MAX™, Becton Dickinson Labware, NY, USA). The aqueous portion in the absorbent paper, mud, or sand was separated in the laboratory using a centrifuge (Kubota 1720). When we could not obtain an aqueous portion using this method, we determined the water retention of each sample by the freeze-drying method. The ions in each dried material were resolved in Milli-Q water at a rate of five or 10 times that of the water retention. The slurry was again centrifuged, and the aqueous portion was removed.

The fluid samples were analyzed by ion chromatography using a PU2080i solvent delivery pump (JASCO, Tokyo, Japan), JASCO CO-2060 column oven, CD-5 conductometric detector (Shodex, Kawasaki, Japan), and JASCO DG-2080-53 online degasser. Separations were achieved on a 4.6 mm ID × 125 mm length fused silica gel column coated with carboxylic polymer (Shodex IC Y-421) using a guard column (Shodex IC YK-G). The mobile phase consisted of water with 5 mM tartaric acid, 1 mM dipicolinic acid, and 24 mM boric acid, with a flow rate of 1.0 ml/min. The column oven temperature was 40 °C, and the detector temperature was 45 °C. The injection volume was 10 μl. Most samples were injected without dilution, but high-concentration samples were diluted 10 times. All samples were filtered through a 0.45-μm filter (GL Sciences, Tokyo, Japan) before injection. Tartaric acid, dipicolinic acid, and boric acid were analytical grade and were purchased from Wako Pure Chemical Industries (Ôsaka, Japan). Distilled water was HPLC grade and was also purchased from Wako Pure Chemical Industries. The concentration of each ion was determined using calibration curves that were obtained from data for five concentrations in the range of 5–100 mg/l. Standards for the Na^+^, K^+^, Ca^2+^, and Mg^2+^ 1 g/l solutions were purchased from GL Sciences. The peak areas of the standards were plotted against the concentrations.

### Electrophysiological experiments

The butterflies used in this experiment were male *P*. *xuthus*, male *P*. *bianor*, and male *P*. *protenor*. All butterflies used in these studies were caught in the field or obtained from the Butterfly House of the Adachi Park of Living Things, Tokyo, Japan. Although *P*. *xuthus* rarely puddles, this species is very common and is representative of the subgenus *Princeps* in Japan. Additionally, the interior structures of the proboscis of this species have been previously shown by Inoue et al. (2009).

The electrical activities of taste receptor neurons in the proboscis sensilla were recorded using the tip-recording method as described in a previous article (Inoue et al. 2009). After anesthetization with ice, a butterfly was fixed right side down in our original experimental chamber using pieces of plastic wrap film. The chamber was set on the platform of a binocular microscope. The proboscis was separated into two galeae with forceps, and the right galea was fixed with adhesive tape in a specific posture to expose the inner surfaces of the right galea on which the sensilla were present. The inner surfaces of the galeae, which were often covered with a viscous substance, were rinsed with purified water. A tapered glass capillary, which was filled with an electroconductive solution as a stimulus material, capped the tip of a given sensillum as both a recording electrode and a stimulating device. The glass capillary was connected to a high-impedance amplifier specialized for tip recording (TastePROBE amplifier, Syntech, Hilversum, The Netherlands) via a platinum wire inserted into the capillary. An indifferent electrode, which was constructed using an electrolytically sharpened tungsten wire, was inserted into the left midleg or left galea and connected to the amplifier. The stimulus solutions used in this experiment were 1 mM NaCl, 10 mM NaCl, 100 mM NaCl, 10 mM KCl, 10 mM CaCl_2_, and 10 mM MgCl_2_. The interstimulus interval was more than 30 s to allow the receptor cells to recover full excitability. Electrical signals were transferred to an A/T compatible computer through the TastePROBE DTP-02 (Syntech) and recorded as a PCM wave file (*.WAV) using Audio Editor in Ulead® Media Studio® Pro (Ulead Systems, Taipei, Taiwan). The signals were printed using original software written with F-BASIC V6.3™ (Fujitsu Middleware Co., Ltd., Tokyo, Japan) on Windows Me, XP, or 7 (Microsoft, Redmond, WA, USA). The response magnitude was shown by the number of impulses, which were counted from 0.13 to 1.13 s after stimulus onset.

## Results

### Morphology

**Fig. 1:**
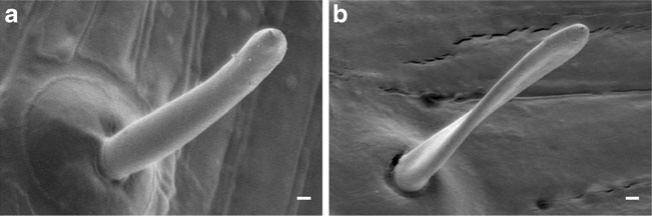
Scanning electron microscope images of the taste sensilla on the inner galea wall of the proboscis in *P*. *bianor* (**a**) and *P*. *protenor* (**b**); *bar* = 10 μm

The proboscises of *P*. *bianor* and *P*. *protenor* were approximately 30 mm in length, and those of *P*. *xuthus* were approximately 20 mm in length. All contact chemosensilla inside the proboscises of both *P*. *bianor* and *P*. *protenor* had single terminal pores and smooth-surfaced side walls (Fig. 1). These characteristics corresponded to those of *P*. *xuthus* (Inoue et al. 2009). The number of sensilla in one galea was approximately 30 in *P*. *bianor* and *P*. *protenor* and approximately 40 in *P*. *xuthus*, as discussed in a previous article (Inoue et al. 2009).

### Behavioral experiments

We observed puddling behaviors by 212 males of eight Japanese *Papilio* species (i.e., *P*. *machaon*, *P*. *xuthus*, *P*. *maackii*, *P*. *bianor*, *P*. *helenus*, *P*. *protenor*, *Papilio macilentus*, and *P*. *memnon*) in our trays at the 28 field experimental sites and two greenhouses. The species and places/dates of this experiment are shown in Table 1, and the list of scores is shown in Table 2. Because the puddling behavior scores were similar between all species, as shown in Table 2, all scores were combined in the subsequent analyses. Statistical results of the behavioral experiments were obtained using Fisher's exact test (*p* < 0.01). In this analysis, Scores of 1 and 2 were treated as one group.

**Table 1:** Places and dates where and when field and greenhouse experiments were performed

Location (prefecture)	(City/village)	Date	Species	Butterfly number	Results obtained
Aomori	Aomori	2009, Jun 20	*bianor*	1	5
	Nishimeya	2009, Jun 21	*maackii*	3	11
Tochigi	Chuuzenji	2007, Aug 25	*maackii*	1	2
		2008, Aug 16	*maackii*	2	8
			*bianor*	1	2
	Kuzuu	2009, Aug 22	*maackii*	2	5
			*bianor*	1	2
			*protenor*	1	4
Tokyo	Hinohara	2009, May 20	*maackii*	6	16
			*bianor*	2	4
			*macilentus*	6	20
		2009, Aug 21	*bianor*	2	7
			*protenor*	1	2
			*macilentus*	3	3
		2010, May 08	*maackii*	1	1
		2010, Jun 10	*maackii*	2	12
			*macilentus*	2	9
		2010, Aug 01	*bianor*	1	1
	Hachiouji	2008, Aug 09	*bianor*	2	4
			*helenus*	1	3
			*protenor*	4	17
			*macilentus*	4	21
		2009, May 14	*macilentus*	3	11
		2009, Aug 01	*macilentus*	1	6
		2010, Aug 01	*protenor*	1	9
			*macilentus*	1	5
Kanagawa	Fujino	2008, Jul 17	*maackii*	1	6
	Yamakita	2007, Aug 05	*maackii*	1	8
			*bianor*	1	9
		2007, Aug 19	*maackii*	2	10
			*bianor*	2	10
		2008, Aug 03	*maackii*	4	22
			*bianor*	4	24
Shizuoka	Suntou	2010, Sep 05	*maackii*	1	4
			*bianor*	1	7
			*protenor*	1	1
Kanagawa	Ôiso	2008, Jul 20	*bianor*	1	1
		2009, Aug 29	*protenor*	1	4
			protenor*	1	1
			*memnon*	2	12
Nagano	Karuizawa	2009, Jun 09	*maackii*	6	14
			*bianor*	1	4
			*macilentus*	1	4
Ishikawa	Senjô	2009, Aug 18	*machaon*	1	7
			*maackii*	4	19
			*bianor*	3	22
		2010, Jun 06	*maackii*	2	5
			*bianor*	1	3
		2010, Aug 23	*machaon*	1	5
			*maackii*	2	7
			*bianor*	3	12
Gifu	Neo	2009, Aug 24	*maackii*	1	7
			*bianor*	6	26
		2010, May 29	*maackii*	2	9
			*bianor*	4	13
			*protenor*	1	2
			*macilentus*	2	11
		2010, Aug 20	*maackii*	3	16
			*bianor*	6	24
			*helenus*	1	6
Gifu	Kasuga	2009, Aug 24	*maackii*	1	4
			*bianor*	2	11
			*protenor*	1	1
			*macilentus*	2	8
Shiga	Yogo	2010, Jun 05	*maackii*	1	7
			*bianor*	3	11
		2010, Aug 21	*maackii*	1	1
			*bianor*	4	12
			*helenus*	2	4
			*macilentus*	1	1
Wakayama	Aridagawa	2009, May 01	*bianor*	1	1
		2009, Sep 04	*xuthus*	1	4
		2010, Jul 18	*xuthus*	1	3
		2010, Aug 09	*xuthus*	5	30
			*protenor*	2	14
		2010, Aug 29	*maackii*	1	6
		2010, Sep 12	*protenor*	2	12
Hyôgo	Shisô	2009, May 05	*maackii*	2	5
			*bianor*	1	4
Okayama	Kamisaibara	2009, Aug 11	*maackii*	1	10
			*bianor*	1	7
Hiroshima	AkiÔta	2009, Sep 06	*bianor*	1	6
		2010, Aug 11	*bianor*	1	3
Shimane	Arifuku	2009, Aug 14	*helenus*	6	31
Fukuoka	Dazai-Fu	2009, Sep 07	*bianor*	2	4
	Wakamiya	2010, Aug 13	*maackii*	1	3
			*bianor*	2	16
			*helenus*	1	1
			*protenor*	5	27
			*macilentus*	1	3
Ôita	Ôita	2009, Sep 11	*helenus*	2	17
			*protenor*	1	6
Kagoshima	Yamagawa	2007, Oct 05	*helenus*	1	1
		2008, Aug 19	*helenus*	1	10
		2008, Sep 21	*helenus*	8	38
			*protenor*	2	9
			*memnon*	2	5
		2009, Sep 24	*helenus*	5	14
Toyosato Insect Museum Green House			*xuthus*	1	4
			*helenus*	3	11
			*memnon*	1	4
Adachi Park of Living Things			*memnon*	1	1
Total				213	895

“protenor*” indicates a female *P*. *protenor*

**Table 2:** Results of the behavioral experiment

Species	Examined Solutions									
	10 mM	10 mM	10 mM	10 mM	1 mM	100 mM	1 M	10 mM	10 mM	10 mM
	NaCl	KCl	CaCl_2_	MgCl_2_	NaCl	NaCl	NaCl	NaNO_2_	NaHCO_3_	NaH_2_PO_4_
*P*. *machaon*	(0, 0, 2)	(1, 0, 0)	(1, 0, 0)	(1, 0, 0)	(1, 0, 0)	(0, 0, 1)	(0, 1, 0)	(0, 0, 2)	(0, 0, 1)	(0, 0, 1)
*P*. *xuthus*	(0, 0, 5)	(2, 0, 0)	(3, 0, 0)	(4, 0, 0)	(0, 2, 1)	(1, 1, 2)	(0, 1, 0)	(0, 0, 5)	(0, 0, 5)	(0, 0, 5)
*P*. *xuthus* ^†^	(0, 0, 1)	(0, 1, 0)	(0, 0, 1)	(1, 0, 0)						
*P*. *maackii*	(0, 0, 28)	(18, 2, 3)	(18, 2, 1)	(20, 0, 0)	(3, 6, 11)	(0, 6, 13)	(4, 9, 5)	(2, 0, 21)	(0, 1, 22)	(0, 1, 22)
*P*. *bianor*	(0, 0, 24)	(23, 2, 0)	(21, 4, 1)	(21, 3, 0)	(4, 7, 17)	(4, 9, 16)	(4, 15, 2)	(0, 0, 25)	(0, 1, 26)	(0, 1, 25)
*P*. *helenus*	(0, 0, 15)	(12, 0, 0)	(8, 2, 0)	(13, 0, 0)	(2, 2, 11)	(1, 0, 13)	(2, 8, 5)	(0, 1, 8)	(0, 0, 11)	(0, 0, 11)
*P*. *helenus* ^†^	(0, 0, 1)	(1, 1, 0)	(1, 1, 0)	(0, 1, 0)		(0, 0, 1)	(0, 1, 0)	(0, 0, 1)	(0, 1, 0)	(0, 0, 1)
*P*. *protenor*	(0, 0, 11)	(10, 3, 0)	(7, 5, 0)	(10, 2, 0)	(2, 4, 5)	(0, 3, 8)	(2, 7, 0)	(0, 0, 10)	(0, 0, 10)	(0, 1, 8)
*P*. protenor*						(0, 0, 1)				
*P*. *macilentus*	(0, 0, 13)	(8, 0, 0)	(8, 1, 0)	(10, 1, 0)	(2, 4, 5)	(0, 0, 10)	(2, 7, 1)	(0, 0, 8)	(0, 0, 10)	(0, 0, 12)
*P*. *memnon*	(1, 0, 1)	(1, 0, 0)	(1, 0, 0)	(2, 0, 0)	(2, 0, 0)	(0, 2, 1)	(1, 1, 0)	(0, 0, 1)	(0, 0, 2)	(0, 0, 2)
*P*. *memnon* ^†^	(0, 0, 1)	(0, 1, 0)	(0, 1, 0)	(1, 0, 0)						
*P*. *memnon* ^††^										(0, 0, 1)
Total	(1, 0, 102)	(76, 10, 3)	(68, 16, 3)	(87, 7, 0)	(16, 25, 50)	(6, 21, 65)	(15, 50, 13)	(2, 1, 81)	(0, 3, 87)	(0, 3, 87)

Puddling scores of eight species of 212 male *Papilio* butterflies and a female *P*. *protenor* in response to test solutions in puddling trays. Numerical values in (**a**, **b**, **c**) are as follows: a, the number of butterflies that were estimated as showing **a** “score 1” behavior to a corresponding test solution; **b**, the number of butterflies that were estimated as demonstrating a “score 2” behavior to a corresponding test solution; **c**, the number of butterflies that were estimated as showing a “score 3” behavior to a corresponding test solution. The scores were high for puddling behaviors to the NaCl solutions in all species of butterflies

“*xuthus* ^†^”; “*helenus* ^†^” and “*memnon* ^†^” indicate that these butterflies were kept in the Toyosato insect Museum Green House

“*memnon* ^††^” indicate that this butterfly was kept in the Adachi Park of living things, Tokyo

All other results were obtained in the field

“protenor*” indicates a female *P*. *protenor*

**Fig. 2:**
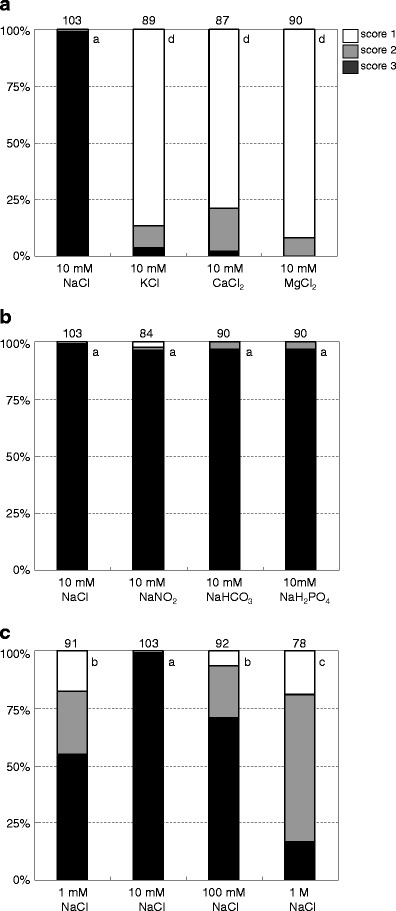
Comparison of taste preferences based on the results of all observed male butterflies. **a** Differences among metal ions; **b** differences among anions; and **c** differences among NaCl concentrations. The *number at the top of each bar* shows the total number of examined butterflies, and the *letters on the right side of each bar* show the grouping results obtained by Fisher's exact test (*p* < 0.01). In this statistical analysis, scores of 1 and 2 were treated as one group. See text for definitions of scores 1, 2, and 3

All butterflies, except for one described below, had scores of 3 for 10 mM NaCl, whereas the scores were lower for other chloride solutions and for those containing different metal ions at the same concentration (Fig. 2a). In contrast, Na^+^ solutions with different species of anions (10 mM NaCl, 10 mM NaNO_2_, 10 mM NaHCO_3_, and 10 mM NaH_2_PO_4_) predominately scored 3 despite the difference in anion species (Fig. 2b). These results indicate that the cation species is the most important factor in selecting a puddling site and that anions do not affect *Papilio* butterflies. Furthermore, the results also illustrate that *Papilio* butterflies prefer Na^+^ solutions for puddling.

Since Na^+^ ions were preferred among the constituent ions in puddling sites, we examined the relationship between Na^+^ concentration and puddling rates. The puddling scores of four different Na^+^ ion concentrations, i.e., 1 mM NaCl, 10 mM NaCl, 100 mM NaCl, and 1 M NaCl, were examined (Fisher's exact test, *p* < 0.01; Fig. 2c). For both 1 mM NaCl and 100 mM NaCl, a score of 3 was the most frequent, and a score of 1 the least frequent. For 1 M NaCl, a score of 2 was the most frequent, and 3 the least frequent. All scores for 10 mM NaCl concentrations except one were scores of 3.

**Fig. 3:**
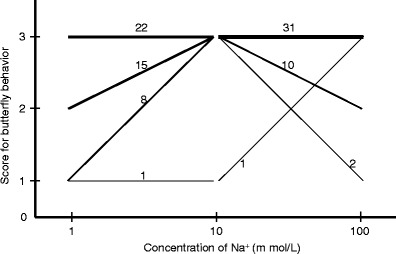
Concentration–response curves for NaCl concentration versus puddling score for each butterfly that exhibited a response to both 1 mM NaCl and 10 mM NaCl and/or 10 mM NaCl and 100 mM NaCl. The width of each line corresponds to the number of butterflies that showed the combination of results for both 1 mM NaCl and 10 mM NaCl or 10 mM NaCl and 100 mM NaCl. *Numbers beside each line* show the numbers of butterflies that displayed the combination of results

Score transition patterns with respect to NaCl concentration were analyzed as shown in Fig. 3. The numbers of butterflies that scored 1, 2, or 3 for 1 mM NaCl and that shifted to score 3 for 10 mM NaCl were 8, 15, and 22, respectively. Thirty-one butterflies were recorded as scoring 3 for both 10 and 100 mM NaCl. The numbers of butterflies scoring 3 for 10 mM NaCl and scoring 1, 2, and 3 for 100 mM NaCl were 2, 10, and 31, respectively. In other words, the fraction of high scores increased with increasing NaCl concentration from 1 to 10 mM NaCl in 23 of 46 individuals, and it decreased from 10 mM to 1 M NaCl in 12 of 44 individuals. One *P*. *memnon* showed an exceptional score pattern, scoring 1 both for 1 and 10 mM NaCl and scoring 3 for 100 mM NaCl.

Puddling by *P*. *machaon*, *P*. *xuthus*, and *P*. *memnon* was rarely observed, especially in the field, even though both *P*. *machaon* and *P*. *xuthus* are very common in Japan. We obtained results for *P*. *machaon* only at Senjô, Ishikawa, and for *P*. *xuthus* only at Aridagawa, Wakayama.

Incidentally, the butterflies that identified and puddled in the trays containing favorable solutions never puddled on the ground, except for two individuals. Additionally, some butterflies that did not locate any favorable solutions in our trays flew away from the experimental site. Moreover, during tray rearrangement, most butterflies flew around the shoulders of the operator or stayed on nearby plants within approximately 10 m of the trays. Soon after completion of the tray rearrangement, the butterflies returned to the experiment site, searched for the favorable solution, and started to puddle immediately. Two exceptional behaviors were shown by one *P*. *maackii* and one *P*. *helenus* that scored 2 or 3 on 1 M NaCl. After puddling in this solution, they displayed abnormal behaviors, such as sipping ground water and cramping their wings after puddling in our trays.

Allegedly, *Papilio* females rarely puddle in the field; however, we observed a female *P*. *protenor* puddling and obtained a behavioral score, which is also shown in Tables 1 and 2. The result was almost identical to that of the males.

### Ion concentration measurements at puddling sites

Table 3 shows the concentrations of four metal ion species in the solutions collected at puddling points and the numbers of *Papilio* butterflies puddling at the sampling sites. The concentrations of metal ions at natural pudding sites were 0.06–5.99 mM for Na^+^, 0.02–11.33 mM for K^+^, 0.10–28.15 mM for Ca^2+^, and 0.02–5.91 mM for Mg^2+^. Metal ion concentrations were quite low at most puddling sites. The concentrations of four cation species varied widely among the sampled solutions. The concentration ratios of the solutions varied, and no particular patterns were observed. The Na^+^ concentrations in the solutions that were sampled from natural puddling sites were less than 1 mM in 48 of 60 samples, even though most of the samples were collected from sites exposed to sunlight on sunny days.

**Table 3:** Concentrations of the four metal ions at the puddling sites

Prefecture	City/village	Date	Status	Concentration (mM)				Butterflies coming to puddle
				Na	K	Ca	Mg	
Aomori	Aomori	2010, Jun 19	in mud^a^	1.88	0.21	1.82	2.10	1
			in mud^a^	3.04	0.39	10.81	2.45	1
	Nishimeya	2010, Jun 20	in sand	1.04	0.06	1.05	1.33	1
			in sand	1.90	0.08	1.00	1.38	1
Tokyo	Hinohara	2010, Jun 10	stream	0.22	0.02	0.15	0.06	2
			stream	0.20	0.02	0.28	0.06	4
			stream	0.24	0.03	0.47	0.08	2
	Hachiouji	2010, Aug 01	stream	0.24	0.05	0.48	0.04	1
			stagnant	3.66	0.29	9.24	4.78	2
			stream	0.29	0.07	0.20	0.09	2
			stream	0.29	0.04	0.51	0.17	2
			in moss	0.28	11.33	28.15	3.74	2
Kanagawa	Fujino	2010, Jul 10	stream	0.20	0.02	0.17	0.08	1
	Ôiso	2010, Jul 18	stagnant	0.42	0.04	0.74	0.37	1
			waste water	0.88	0.13	0.40	0.20	1
Shizuoka	Suntou	2010, Aug 07	stream	0.23	0.05	0.32	0.24	5
			stream	0.26	0.07	0.82	0.44	5
			stream	0.32	0.18	1.32	0.62	5
Nagano	Karuizawa	2010, Jul 06	in mud	0.39	0.22	1.06	0.30	1
			stream	0.14	0.02	0.24	0.10	1
		2012, Aug 24	in mud^a^	3.79	0.40	10.48	1.42	1
Ishikawa	Senjou	2010, Aug 23	stream	0.32	0.03	0.37	0.15	1
			stream	0.32	0.03	0.26	0.08	1
			in mud	0.74	0.11	2.71	0.59	1
			in mud^a^	2.47	0.26	2.24	2.05	20
			in sand^a^	0.84	0.06	1.55	0.54	5
			in sand^a^	5.99	0.95	11.29	5.91	3
Gifu	Neo	2010, Aug 24	stream	0.32	0.02	0.10	0.04	5
			stream	0.12	0.03	0.49	0.12	5
			stream	0.16	0.03	0.73	0.18	5
			stream	0.84	0.10	0.52	0.18	20
			in mud^a^	0.06	1.28	4.16	1.82	10
		2011, Jun 04	in ash^a^	3.63	0.56	1.78	5.08	20
Shiga	Yogo	2010, Aug 21	stream	0.35	0.03	0.46	0.07	5
			stream	0.51	0.05	0.73	0.09	5
			stream	0.38	0.07	0.92	0.14	2
			stream	2.30	0.68	1.75	0.69	10
			stream	0.21	0.05	0.35	0.12	4
Wakayama	Kibi	2010, Aug 09	stream	0.17	0.03	0.16	0.05	1
			stream	0.41	0.05	0.20	0.06	2
			in mud^a^	2.50	0.72	13.74	3.94	5
	Shimizu	2010, Sep 12	in sand^a^	0.34	0.06	1.55	0.33	1
			in mud^a^	0.69	1.23	12.27	3.13	2
	Kanaya	2010, Aug 29	stream	0.96	0.09	0.55	0.06	5
			stream	0.18	0.10	0.80	0.23	2
Hyôgo	Shisou	2010, May 03	stream	0.19	0.02	0.16	0.05	1
			stream	0.16	0.03	0.31	0.04	1
Okayama	Takahashi	2009, Aug 12	stream	0.22	0.10	0.13	0.02	1
Hiroshima	AkiÔta	2010, Aug 11	in litter^a^	1.04	2.96	12.31	2.06	3
Shimane	Goutsu	2010, Jun 16	stream	0.52	0.04	0.21	0.13	1
			stream	0.40	0.10	0.25	0.10	1
Yamaguchi	Boushû	2010, Aug 15	stream	0.19	0.10	0.17	0.07	1
			stream	0.35	0.05	0.16	0.03	1
Fukuoka	Wakamiya	2010, Aug 13	in mud^a^	1.10	0.87	6.44	1.68	1
			in mud^a^	0.79	0.34	0.38	0.18	10
			stream	0.40	0.08	0.42	0.15	5
Ôita	Ôita	2010, Jul 21	stream	0.40	0.02	0.41	0.42	4
			stream	0.34	0.02	0.22	0.12	5
Kagoshima	Yamagawa	2010, Aug 27	stream	0.65	0.07	0.45	0.22	20
			stream	0.27	0.25	0.61	0.19	20
			stream	0.54	0.49	0.60	0.22	1

^a^These materials were obtained by scraping away the surface exposed to the air

### Electrophysiological experiments

**Fig. 4:**
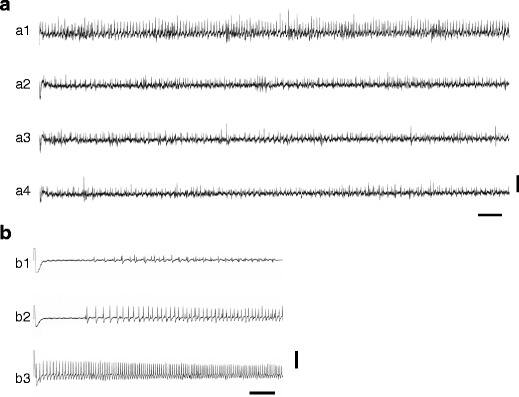
Representative impulse responses to solutions with four different species of metal ions (**a**) and three concentrations of NaCl (**b**). Responses were recorded from the two different sensilla in the proboscis of a male *Papilio xuthus* taken from Toyohashi, Aichi Prefecture, on July 26, 2007. *a1* 10 mM NaCl, *a2* 10 mM KCl, *a3* 10 mM CaCl_2_, *a4* 10 mM MgCl_2_, *b1* 1 mM NaCl, *b2* 10 mM NaCl, *b3* 100 mM NaCl. *Vertical scale bar* in **a** = 0.1 mV; **b** = 0.5 mV; *horizontal scale bar* = 0.1 s

Impulse responses to salt solutions were recorded from contact chemosensilla inside the proboscis as follows: 38 sensilla in 15 *P*. *xuthus*, 25 sensilla in nine *P*. *bianor*, and seven sensilla in nine *P*. *protenor*. When stimulated with 10 mM NaCl, one type of impulse was dominantly recorded from the sensillum on the proboscis of male *P*. *xuthus* with the tip recording method shown in Fig. 4 (a1). Impulses were discharged immediately after the stimulus onset and then increased gradually in frequency. Thereafter, the impulse frequencies became constant over a few seconds. In recordings taken from three sensilla of *P*. *xuthus* and three sensilla of *P*. *bianor*, impulses showed a delayed discharge after stimulus onset, but the delay time became shorter, and impulse frequency became higher with greater concentrations of NaCl (Fig. 4 (b1–b3)). Responses to 10 mM KCl were recorded from about a dozen sensilla of *P*. *xuthus*, *P*. *bianor*, and *P*. *protenor* (Fig. 4 (a2)). Some of these responses were similar to the responses to 10 mM NaCl with respect to impulse size and the time course of the response. In contrast, the impulse responses to 10 mM CaCl_2_ and 10 mM MgCl_2_, which were recorded from the same sensillum, showed a relatively lower impulse height and were discharged irregularly (Fig. 4 (a3 and a4)).

**Fig. 5:**
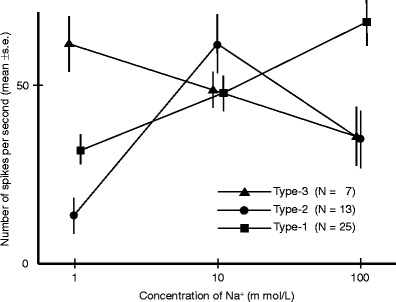
Concentration–response relationships for NaCl concentration versus electrophysiological response recorded from the taste sensilla on the inner galea wall of the proboscis of *P. xuthus*, *P*. *bianor*, and *P*. *protenor*. The number of spikes was counted during the period from 0.13 to 1.13 s after contact with the recording electrode. Values are mean ± SE. See text: types 1–3

We analyzed the relationship between the response magnitude and NaCl concentration in cases where the impulse frequency could be clearly quantified across a NaCl concentration range of 1 to 100 mM (Fig. 5). We identified three different patterns based on this relationship. The response magnitudes increased with increasing NaCl concentrations in 25 neurons (type 1), were largest at 10 mM NaCl in 13 neurons (type 2), and decreased with increasing concentrations of NaCl in seven neurons (type 3). These three types of receptor neurons were present in different sensilla of single butterflies.

## Discussion

We obtained behavioral and electrophysiological results necessary to resolve the inducing factors for butterfly puddling behavior and also obtained morphological and chemical empirical data to support the behavioral and electrophysiological results. In the behavioral experiments, most butterflies had scores of 3 for solutions of 10 mM Na^+^ (Table 2, Fig. 2a, c). Fluctuations in pH caused by weak acids (i.e., NO_2_^−^, HCO_3_^−^, and H_2_PO_4_^−^) did not affect the initiation of puddling behavior for the 10 mM Na^+^ solution (Table 2, Fig. 2b). Only nine butterflies scored 1 or 2 for the 10 mM Na^+^ solutions. Most of these butterflies were discouraged from visiting the trays by many other butterflies that had already puddled there. Thus, they appeared to be disrupted when attempting to puddle, which might be the reason for their low scores. One *P*. *memnon* male had a distinctive response, scoring 3 to 100 mM NaCl and 1 to both 1 and 10 mM NaCl. He came to our experimental site alone; thus, this behavior was not the result of competition with other butterflies. These results indicate that puddling behaviors are initiated by Na^+^ ions and are not affected by anions contained in the Na^+^ solutions. Furthermore, the relationship between the puddling score and NaCl concentration indicates that of the tested concentration range of 1 mM to 1 M, *Papilio* butterflies mostly prefer 10 mM NaCl.

In puddling responses to other cation species, some butterflies scored 2 or 3 to K^+^ and Ca^2+^ and scored 2 to Mg^2+^. Even if the first puddling event at the K^+^ or Ca^2+^ tray was scored as 2 or 3, the second or subsequent puddlings at the 10 mM K^+^, 10 mM Ca^2+^, 10 mM Mg^2+^, or 1 M Na^+^ trays were scored as 1 after the 10 mM Na^+^ tray was identified. These behavioral experiments showed that puddling is strongly induced by Na^+^ in *Papilio* butterflies and is not induced by other cations. Our interpretations mentioned above are mostly consistent with those of Kitaoka (1950); Kuwabara (1951); Arms et al. (1974), and Beck et al. (1999), although those studies differed in butterfly species and/or experimental bases of the behavioral analysis.

In the experiment using HPLC for measurements of ion concentrations at puddling sites in the field, the concentrations of K^+^, Ca^2+^, and/or Mg^2+^ in samples from many puddling sites were found to be higher than that of Na^+^. Thus, cations other than Na^+^ probably do not affect the induction of puddling behaviors in natural solutions. The butterflies that identified the Na^+^-dense solutions in our trays did not puddle in natural sites, and they returned to our trays to puddle during our experiments, with only two exceptions. Na^+^ concentrations at all sites measured in this experiment were less than 10 mM, and butterflies might select sites of more concentrated Na^+^ for puddling in natural environments.

The electrophysiological experiments that recorded impulses from the sensilla inside the proboscis revealed some differences in the characteristics of receptor neurons with regard to their responses to cations as well as to Na^+^ concentrations. The majority of receptor neurons were excited by NaCl but not by CaCl_2_ or MgCl_2_. These neurons, which discharged impulses immediately after stimulus onset (Fig. 4a), appear to be important in the inducement of puddling behavior. Although whether these receptor neurons respond to KCl is unclear, this does not seem to be an issue in the field, as K^+^ concentrations in puddling sites were generally lower than those of Na^+^. When stimulated by MgCl_2_ and CaCl_2_, small and irregularly discharged impulses were recorded from the sensilla housing this type of receptor neuron. We suggest that these impulses belong to receptor neurons that differ from those that respond to NaCl.

Some neurons showed discharge impulses that were delayed relative to the stimulus onset in response to lower concentrations of NaCl (Fig. 4 (b1 and b2)). Furthermore, as the stimulus strength increased, the impulse frequency increased, and the delay time was reduced. This response pattern might make the butterflies react more quickly to stronger stimuli. Similar delayed patterns of impulse discharge were sometimes observed in response to 10 mM CaCl_2_ and 10 mM MgCl_2_ when we recorded from the same sensilla. Therefore, we infer that these sensilla are a different type from the major sensilla described above. Further investigation is needed to identify sensillum types. The concentrations of Mg^2+^ and Ca^2+^ was higher than that of Na^+^ at some puddling sites in the field, and puddling occurred at such sites. Thus, we infer that Mg^2+^ and Ca^2+^ do not affect puddling occurrence in the field.

The dose–response pattern to Na^+^ was quite different from that to sucrose (Inoue et al. 2009), whereas the pattern of type 2 sensilla shown in Fig. 5 corresponded to the behavioral pattern of many butterflies in Fig. 3, whereby higher concentrations of Na^+^ in solutions reduced the number of butterflies that preferred such solutions. Therefore, Na^+^ concentrations can be too high, and this may relate to the rarity of puddling on seawater (Pola and García-París 2005). Actually, as described in the “Results”, at least two butterflies showed abnormal behavior after sipping the 1 M NaCl solution. In humans, excessive intake of Na^+^ can cause many diseases such as hypertension. There may be some “safety system” related to the detection of Na^+^ concentrations.

Our current experiments also prompted at least three other novel questions. One is whether a relationship is present between proboscis length and the number of contact chemosensilla within it. The proboscis lengths of male *P*. *bianor* and *P*. *protenor* nearly corresponded with those of females of some species of Papilionini butterflies, including *P*. *xuthus*, *P*. *bianor*, and *P*. *protenor*, which were examined by Watanabe (1983). *P*. *bianor* and *P*. *protenor* have about 30 sensilla on one galea of the proboscis, and *P*. *xuthus* has about 40 sensilla (Inoue et al. 2009). In contrast, *P*. *xuthus* clearly has a shorter proboscis than both *P*. *bianor* and *P*. *protenor*. Thus, the number of sensilla is not related to body size and proboscis length among these *Papilio* species. We should further clarify this relationship using various other *Papilio* species. The second question is why the frequency of puddling behavior differs among different species. Although puddling behaviors by *P*. *machaon* and *P*. *xuthus* were rarely observed in the field, their distribution patterns of puddling scores were almost identical to those of other *Papilio* species in our field experiments. Furthermore, we found no fundamental differences in the electrophysiological responses of the proboscis sensilla of *P*. *xuthus* and those of *P*. *bianor* and *P*. *protenor*, in which puddling behaviors were frequently observed in the field. Whether these differences might be related to geology, host plant selection, and/or other factors remains unknown. The third question is why the different types of dose response patterns for Na^+^ were recorded from the proboscis sensilla in Fig. 5. An unknown system that modifies sensitivity to Na^+^ might be present.

Insect contact chemosensilla typically have four or five receptor neurons at most. We recorded the responses of sucrose receptor neurons in the proboscis sensilla of butterflies, and they showed that sucrose receptors are surely responsible for sucking nectar from flowers (Inoue et al. 2009). Our present experiments showed that moderate concentrations of Na^+^ solutions were preferred by Japanese *Papilio* butterflies and that the proboscis sensilla housed Na^+^ receptor neurons to detect various concentrations of Na^+^. Given the measured Na^+^ concentrations in the puddling sites in the natural field, we showed that Japanese *Papilio* butterflies puddle using Na^+^ detected and measured in concentration by the contact chemosensilla in the proboscis.

## Supplemental pictures

Butterflies coming to wet riverbed. Kaeng Krachan, Thailand. 02 May 2012. ESM 1 (BMP 900 kb)
ESM 2 (BMP 900 kb)
