# Supplementary figures and images for: Japanese Papilio butterflies puddle using Na+ detected by contact chemosensilla in the proboscis
Source: Naturwissenschaften. 2012 Nov 9;99(12):985–98. doi: 10.1007/s00114-012-0976-3 (PMC3505521; doi:10.1007/s00114-012-0976-3)

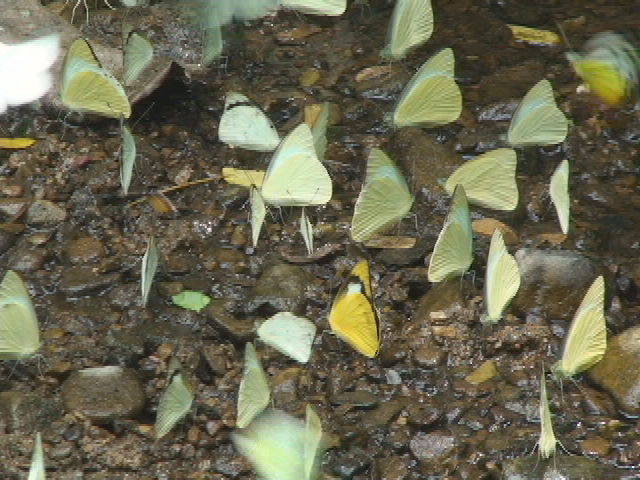

Supplement: ESM 1 — (BMP 900 kb) [file 114_2012_976_MOESM1_ESM.bmp]

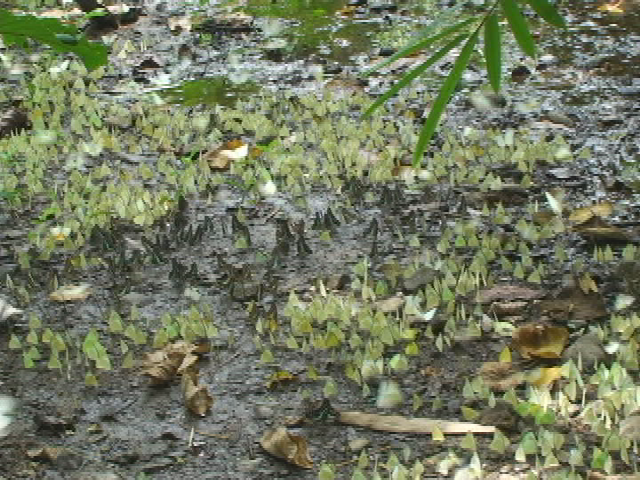

Supplement: ESM 2 — (BMP 900 kb) [file 114_2012_976_MOESM2_ESM.bmp]
